# Analysis and Implementation of Controlled Semiconductor Switch for Ultra-Wideband Radar Sensor Applications

**DOI:** 10.3390/s23177392

**Published:** 2023-08-24

**Authors:** Patrik Jurik, Miroslav Sokol, Pavol Galajda, Milos Drutarovsky

**Affiliations:** Department of Electronics and Multimedia Telecommunications, Technical University of Košice, 042 00 Kosice, Slovakia; patrik.jurik@tuke.sk (P.J.); miroslav.sokol@tuke.sk (M.S.); pavol.galajda@tuke.sk (P.G.)

**Keywords:** ultra-wideband, UWB, SPDT, switch, ASIC, SoC, SiP

## Abstract

All ultra-wideband (UWB) sensor applications require hardware designed directly for their specific application. The switching of broadband radio frequency and microwave signals is an integral part of almost every piece of high-frequency equipment, whether in commercial operation or laboratory conditions. The trend of integrating various circuit structures and systems on a chip (SoC) or in a single package (SiP) is also related to the need to design these integrated switches for various measuring devices and instruments in laboratories, paradoxically for their further development. Another possible use is switching high-frequency signals in telecommunications devices, whether mobile or fixed networks, for example, for switching signals from several antennas. Based on these requirements, a high-frequency semiconductor integrated switch with NMOS transistors was designed. With these transistors, it is possible to achieve higher integration than with bipolar ones. Even though MOSFET transistors have worse frequency characteristics, we can compensate them to some extent with the precise design of the circuit and layout of the chip. This article describes the analysis and design of a high-frequency semiconductor integrated switch for UWB applications consisting of three series-parallel switches controlled by CMOS logic signals. They are primarily intended for UWB sensor systems, e.g., when switching and configuring the antenna MIMO system or when switching calibration tools. The design of the switch was implemented in low-cost 0.35 µm SiGe BiCMOS technology with an emphasis on the smallest possible attenuation and the largest possible bandwidth and isolation. The reason for choosing this technology was also that other circuit structures of UWB systems were realized in this technology. Through the simulations, individual parameters of the circuit were simulated, the layout of the chip was also created, and the parameters of the circuit were simulated with the parasitic extraction and the inclusion of parasitic elements (post-layout simulations). Subsequently, the chip was manufactured and its parameters were measured and evaluated. Based on these measurements, the designed and fabricated UWB switch was found to have the following parameters: a supply current of 2 mA at 3.3 V, a bandwidth of 6 GHz, an insertion loss (at 1 GHz) of −2.2 dB, and isolation (at 1 GHz) of −33 dB, which satisfy the requirements for our UWB sensor applications.

## 1. Introduction

Switching of ultra-wideband (UWB) radio frequency and microwave signals is essential to almost every high-frequency (HF) device, whether in commercial operation or laboratory conditions. Related to the trend of integrating various circuit structures on system on chip (SoC) is the need to design integrated switches for various commercial and measurement HF devices as well as instruments in laboratories, paradoxically for their further development. Hence, selecting the appropriate switch is extremely important for successful RF applications. Switch requirements can vary greatly even within a single system, such as, for example, switching and configuring the antenna MIMO system or when switching calibration tools. When equipped with the knowledge of available RF switch solutions, application requirements, and performance tradeoffs, engineers can effectively implement their designs and meet today’s advanced systems demands. Covering all possible system considerations and applications is beyond the scope of this article, therefore as an example some important and interesting applications will be introduced:

- In telecommunications: The RF switches in cellular networks are essential components in cell towers and base stations, enabling efficient switching between different frequency bands and antennas [1,2,3]. The RF switches in satellite and aerospace communication help route signals in communication systems, allowing for flexible control of downlink and uplink frequencies [4].
- In wireless Wi-Fi and Bluetooth communication systems: The RF switches are used to manage signal paths between antennas and transceivers, improving connectivity and signal quality.
- In fiber optic communication: The RF switches can be used to modulate optical signals for efficient data transmission.
- In defence: High-frequency RF switches are crucial in radar systems for rapid switching between transmission and reception modes, enhancing target detection capabilities, and the RF switches also play a role in electronic countermeasure systems by managing signal routing to disrupt or jam enemy communication systems in scenarios of electronic warfare.
- In medical imaging (for magnetic resonance imaging, ultrasound systems, etc.): The RF switches can help manage signal paths, enhancing image quality and reducing interference, or are used to switch between different transducers and frequencies for diagnostic imaging.
- In the automotive industry: The RF switches are used in automotive radar systems (radar-based driver assistance systems) for collision avoidance, adaptive cruise control, and other safety features.
- In industrial and manufacturing: The RF switches can be used in non-destructive testing and quality control processes, such as material analysis and defect detection [5,6,7,8].
- In process control: The RF switches can help with managing communication and control signals in industrial automation systems [9].
- In research and development: The RF switches are used in scientific instruments for signal routing and testing, such as in spectrum analyzers, signal generators, and calibration systems.

The presented switch has been analyzed and designed to test its capabilities, future applications, and its integration in SoC with other circuits developed for UWB applications, especially for M-sequence based radars [10]. These pseudo-noise radars have been used in many applications, like nondestructive testing tasks in industrial and civil engineering [5], non-invasive medicine diagnostics, and vital sign detection in medical engineering [11], indoor person localization, search and rescue operations [12], and many others. For these applications, a lot of ASIC circuits have been designed and implemented, such as in [6,13,14,15].

In the case of designing electronic switches, the choice between MOSFETs (metal-oxide-semiconductor field effect transistors) and bipolar junction transistors (BJTs) involves careful consideration of various trade-offs. Each type of transistor has its advantages and disadvantages, and the selection depends on factors such as power consumption, speed, voltage handling capabilities, and the intended application. Therefore, in the next section, we introduce some of the trade-offs between these two transistor types in switch design.

The NMOS transistors (MOSFET transistors with channel type-n) can switch the low voltages and they may have problems handling high voltage levels efficiently. The BJTs often have better voltage-handling capabilities compared to NMOS transistors. However, the NMOS transistors are generally more power-efficient in terms of static power consumption, as they have negligible current flow when turned off. On the other hand, the bipolar transistor’s dynamic power consumption can be lower due to faster switching. The NMOS transistors can switch relatively quickly due to their relatively small channel length and low on-resistance. However, they might not be as fast as some bipolar transistors, especially in high-frequency applications. In the case of the threshold voltage, the NMOS transistors require a positive gate-source voltage to turn on, typically around 0.5 V to 1.5 V, while BJTs can operate with very low base-emitter voltage levels. The higher threshold voltage might limit the use of the switch in low-voltage applications. The BJTs are less susceptible to noise due to their current-controlled operation, but the NMOS transistors can be cost-effective due to their compatibility with CMOS processes. The bipolar transistors are often more stable in performance across a wider temperature range. In conclusion, the choice between NMOS and bipolar transistors in switch design depends on the specific requirements of the application. The NMOS transistors are generally more suitable for low-power and digital applications, while bipolar transistors excel in high-speed and high-power scenarios. Because NMOS transistors are symmetrical and thus the $I_{D}-V_{\mathrm{DS}}$ characteristics pass directly through the origin, the NMOS transistors are more suitable for analog switches, while bipolar transistors are asymmetrical with offset voltage $V_{\mathrm{CEoff}}$.

The last, and perhaps most important, difference between the current–voltage characteristics of the two devices concerns the input current into the control terminal. While at low frequencies the gate current of the NMOS is practically zero and the input resistance looking into the gate is practically infinite, the BJT draws a base current IB that is proportional to the collector current. The finite base current and the corresponding finite input resistance looking into the base comprise a definite disadvantage of the BJT in comparison to the NMOS. Thus, as was mentioned earlier, the NMOS provides an excellent implementation of a switch, a fact that has made CMOS technology capable of realizing a host of analog circuit functions that are not possible with bipolar transistors.

It can thus be seen that each of the two transistor types has its own distinct and unique advantages. Bipolar technology has been extremely useful in the design of very high-quality general-purpose circuit building blocks, such as op-amps. On the other hand, CMOS, with its very high packing density and its suitability for both digital and analog circuits, has become the technology of choice for the implementation of very large-scale integrated circuits. Nevertheless, the performance of CMOS circuits can be improved if the designer has available (on the same chip-SoC) bipolar transistors that can be employed in functions that require their high transconductance and excellent current-driving capability. A technology that allows the fabrication of high-quality bipolar transistors on the same chip as CMOS circuits is called BiCMOS.

The decision should be made based on a careful evaluation of the trade-offs discussed above and an understanding of how they align with the desired functionality and performance of the switch circuit.

Based on these requirements and the analysis, an interesting and useful controlled semiconductor switch for ultra-wideband radar sensor applications with NMOS transistors, implemented in low-cost 0.35 µm SiGe BiCMOS technology, was designed. Although metal-oxide-semiconductor field-effect transistors (MOSFETs) have worse frequency characteristics, we can compensate them to some extent by proper circuit and chip design. The priority was to achieve the lowest possible signal attenuation and the largest possible frequency bandwidth that the switch could handle. We will use several options to switch high-frequency signals and achieve the aforementioned desired characteristics. For example, improving an already used circuit structure for a different manufacturing technology. The design of switches can be realized by different technologies, ranging from a mechanical switch to micro-electromechanical elements (MEMS) [16], to a diode [17] or transistor switch integrated into a chip [18,19]. In the case of the integrated transistor switch, CMOS transistors are most commonly used as switching elements.

**The main advantages of CMOS transistors used as switches are:**

- Separation of control and switching signals due to isolated gate.
- Elimination of the mechanical elements of the switch, resulting in a small size, high degree of integration, high reliability, and good switching speed.
- Relatively inexpensive mass production with well-mastered technology.
- Easy integration on an SoC with control logic and other circuitry.
- Low power consumption resulting from voltage control of switching elements.
- Ability to switch both positive and negative voltages.

**However, we must also mention the disadvantages, which mainly include:**

- Higher resistance and parasitic capacitance as well as associated insertion loss, and lower frequency bandwidth.
- Worse isolation and crosstalk at higher frequencies.
- Greater power limitation and associated signal distortion at higher powers.

The simplest circuit enabling analog signal switching in CMOS technology is the serial single pole double through the Transmit/Receive (SPDT T/R) switch [20]. Each switched channel contains one MOSFET transistor connected in series to the signal path. The transistors are controlled by a logic structure that controls them using a control voltage. This switch has relatively low insertion loss and few parasitic elements. A series-parallel switch was created by modifying such a serial switch, i.e., by adding additional transistors connected in parallel. This modification will increase the isolation in the open state of the switch [21]. The series-parallel switches are divided into [22]:

- Absorptive—in the open state, a matched impedance is connected to the input instead of the output channel, which absorbs the signal;
- Reflective—in the open state, the switch behaves as a short or open circuit from the source’s point of view, i.e., the signal is reflected to the source.

A differential SPDT T/R switch [20] is used to switch differential signals. Such a switch can be constructed from two series of switches that are mirrored with respect to each other. Differential connections are often used because of better performance characteristics. In addition, the differential topology represents greater immunity to interference, provides higher linearity, lower substrate noise, and lower sensitivity to supply voltage variations. A CMOS switch [20] can switch signals whose value reaches close to the supply voltage value. In this case, a complementary pair of MOSFET transistors is used instead of one or several NMOS transistors. The resulting resistance of such a switch is almost independent of the input voltage.

By modifying the series-parallel T/R switch topology can also realize an asymmetrical SPDT T/R switch [20]. For example, by omitting one transistor and replacing the other with a switched impedance. The switched impedance contains additional inductances. The quality factor of the implemented inductor affects the performance and frequency bandwidth, and the implementation of the inductance in integrated form is quite challenging.

Another solution offered is a switch with a travelling wave [20]. Such a circuit is a series connection of inductors to which transistors are connected in parallel. Similar to the switched impedance circuit, this solution is also difficult to implement in integrated form due to the use of inductance.

The next section of this paper describes the topology of the proposed semiconductor integrated switch with NMOS transistors. The third section focuses on the description of the circuit design, along with its simulations and the design of the chip layout. The design is implemented in the available low-cost 0.35 µm BiCMOS technology. The reason for choosing this technology was also that other circuit structures of UWB Systems were realized in this technology [13] as well. Finally, this paper concludes with an evaluation of the results.

## 2. The Technology Used for the Integrated UWB Switch

Today’s MOSFET switches are a challenge in many applications because their cutoff frequencies and breakdown voltages are constantly improving. Each technology has advantages and disadvantages that can be exploited for different applications for an optimal solution.

### 2.1. Model of the MOSFET Transistor for SiGe BiCMOS Technology

The metal-oxide-semiconductor field-effect transistors (MOSFETs) have become an increasingly essential part of integrated circuits because they enable high density and low power dissipation. Bipolar transistors also provide many advantages in integrated circuits. For example, the gain factor per unit current is typically higher in bipolar transistors than in MOSFET ones [23]. Thus, in systems where both analog and digital integrated circuits are required, bipolar technology is more suitable for analog integrated circuits, and metal-oxide-semiconductor technology (MOS) is more suitable for digital integrated circuits. One way to achieve these goals is to use BiCMOS technology, which supports on-chip implementation of bipolar and MOS transistors, allowing for great design flexibility. However, the MOS process of chip manufacturing is cheaper than the bipolar fabrication process. As a result, the research and use of MOS transistors would be intended for analog designs in electronic applications [24].

The basic element of the proposed high-frequency semiconductor switch described in this paper is an NMOS transistor. Its design has been implemented in a low-cost 0.35 µm SiGe BiCMOS technology. For basic design and calculations, it is convenient to use a model of the transistor that accurately represents its behavior in the circuit as well as its properties in the implemented fabrication technology. It should be noted that these models should not be very complex to facilitate computations. More on the description of the operation and the corresponding working conditions of MOSFET transistors, with channel type-n (NMOS) and with channel type-p (PMOS) in the enhancement mode, is given in [23,24].

**Figure 1:**
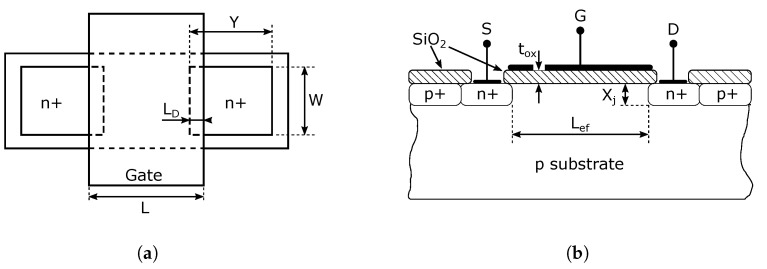
Structure of NMOS transistor. (**a**) Top view of a typical NMOS transistor. (**b**) Cross-section of a typical NMOS transistor.

Figure 1 shows a cross-section and top view of a typical NMOS transistor operating in the enhancement mode. The gate width, denoted as *L*, may extend beyond the source and drain region (see Figure 1a). If we know the value of the width *L* and the value of $L_{D}$, the effective channel length can be calculated according to the following relation [25]:

(1) $$L_{\mathrm{ef}}=L-2L_{D}.$$

The surfaces of the source and the drain are the same, which shows the symmetry of the MOSFET transistor structure. Typical $L_{D}$ values are given in the table of specific parameters and parasitic capacitances (Table 1). The diffusion regions of the source and drain have a width denoted by *W*, a length represented by *Y*, and a depth indicated by $X_{j}$. Both regions are surrounded by a p+ type semiconductor on each side to prevent the formation of any unwanted conduction channels between two adjacent n+ diffusion regions in the case of integrated circuit design.

### 2.2. Parasitic Capacitances in BiCMOS Technology

Based on the physical structure, pinout arrangement, and other influences, it can be argued that in terms of high frequencies, the MOSFET transistor has parasitic capacitances that make worse the characteristics of high-frequency circuits created from these transistors. These parasitic capacitances must have the smallest possible value allows to increase these circuits’ switching speed and bandwidth. The capacitances in the MOSFET’s structure are distributed, and their exact calculation is quite difficult to determine. Using a simple approximation, we can obtain a model and values of the parasitic capacitances that can be used to analyze the transistor behavior in the high-frequency signal domain.

The fundamental capacitances that most affect the transistor behavior for RF signals can be divided into two main groups:

- The gate capacitance effect, characterized by the $C_{\mathrm{ox}}$ parameter;
- The junction capacitances between drain (D)-substrate also called the “bulk” or the body (B) and source (S)-substrate (B) semiconductor junctions.

**Figure 2:**
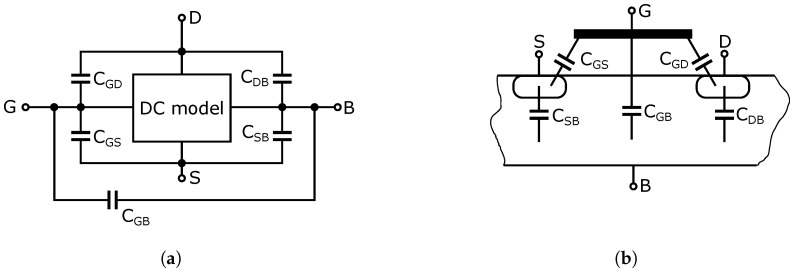
Connecting the parasitic capacitances in an NMOS transistor (**a**) schematic, (**b**) NMOS transistor parasitic capacitance model.

The transistor behavior and the two main capacitance effects can be modelled by using a direct current (DC) NMOS transistor model and connecting the capacitances between the four terminals of the transistor as shown in Figure 2. In this way, we obtain five main capacitances: $C_{\mathrm{GS}}$, $C_{\mathrm{GD}}$, $C_{\mathrm{GB}}$, $C_{\mathrm{SB}}$ and $C_{\mathrm{DB}}$, where the indexes denote the terminals of the transistor [23,24,25].

**Figure 3:**
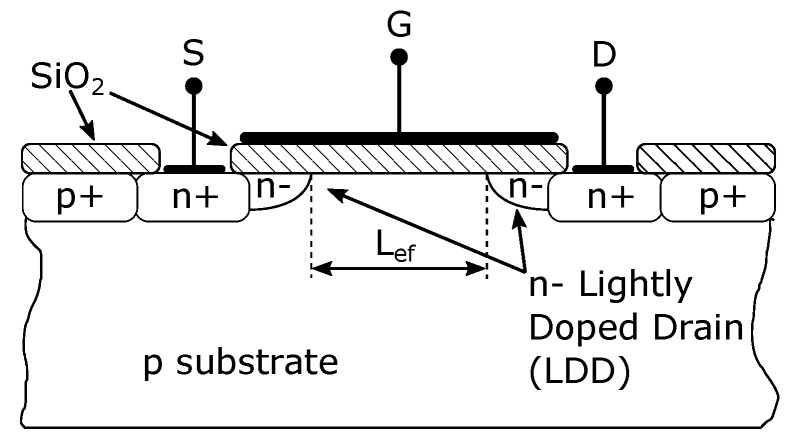
Cross section of NMOS with lightly doped drain (LDD) structure.

The gate capacitance effect is characterized by $C_{\mathrm{GSov}}$ and $C_{\mathrm{GDov}}$ (ov- overlap) and $C_{\mathrm{GD}}$, $C_{\mathrm{GS}}$ and $C_{\mathrm{GB}}$ capacitances [25,26]. Parasitic capacities of $C_{\mathrm{GSov}}$ and $C_{\mathrm{GDov}}$ can arise in the case of overlapping gates over the drain and source regions or in low doped drain (LDD) technology (see Figure 3) [27]. Assuming that the NMOS transistor has a symmetrical structure, that is, the drain and source are geometrically identical, the overlapping capacitances can be calculated from the following equations [23,24,25]:

(2) $$C_{\mathrm{GSov}}=C_{\mathrm{ox}}WL_{D}$$

(3) $$C_{\mathrm{GDov}}=C_{\mathrm{ox}}WL_{D}.$$

The calculation of the $C_{\mathrm{ox}}$ capacity is given by the relation:

(4) $$C_{\mathrm{ox}}=\varepsilon_{ox}/t_{ox},$$

where $\varepsilon_{ox}$ is the permittivity of gate oxide and $t_{ox}$ is the thickness of the gate oxide. The overlapping parasitic capacitances are voltage independent. The capacity for LDD technology is mostly directly specified in the manufacturer’s datasheets. By applying LDD technology, the capacitances are reduced by approximately half compared to the original structure.

The capacitances that result from the interaction between the electrodes are denoted as $C_{\mathrm{GD}}$, $C_{\mathrm{GS}}$ and $C_{\mathrm{GB}}$. These capacitances change their magnitude depending on the operating mode of the MOSFET transistor and are voltage-dependent. When the transistor operates in the cutoff state, no conductive channel is formed, the capacitances $C_{\mathrm{GD}}$ and $C_{\mathrm{GS}}$ become zero, and the capacitance $C_{\mathrm{GB}}$ then has the highest value determined from the relation:

(5) $$C_{\mathrm{GB}}=C_{\mathrm{ox}}WL.$$

A transistor operating in triode region has a conducting channel formed along its entire length from the source to the drain. The thickness of the channel at the drain only changes significantly as the saturation mode is approached. The capacitance $C_{\mathrm{GB}}$ is zero in this case, and the capacitances $C_{\mathrm{GD}}$ and $C_{\mathrm{GS}}$ can be calculated from equation:

(6) $$C_{\mathrm{GS}}=C_{\mathrm{GD}}=\frac{1}{2}C_{\mathrm{ox}}WL.$$

The last mode of the transistor in which the gate capacitances change their values is the saturation region. The $C_{\mathrm{GB}}$ capacity is zero in the saturation region. In the saturation region, the channel is not conductive along its entire length from the drain to the source. The channel ceases to be conductive beyond the so-called pinch-off point. At the drain, the conductive channel disappears, which means that the value of the $C_{\mathrm{GD}}$ capacity will be the same as in the cutoff region, i.e., zero. The presence of a conducting channel at the source creates a capacitance similar to that in the ohmic region. The value of this capacity can be approximately determined from the following relationship [25]:

(7) $$C_{\mathrm{GS}}\approx \frac{2}{3}C_{\mathrm{ox}}WL.$$

**Figure 4:**
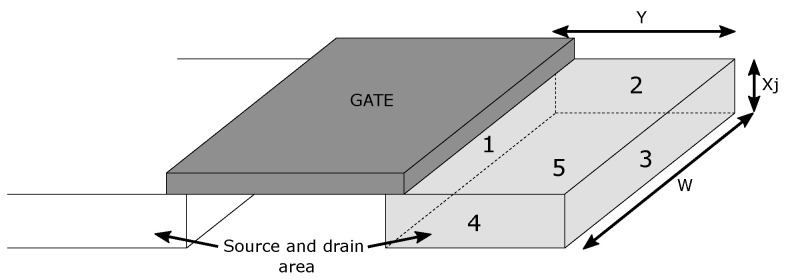
View of the drain and source area with the junction surfaces marked.

Junction capacities create another capacity effect. These capacitances arise between the semiconductor drain-body and source-body junctions [25,28]. The three-dimensional shape of the n+ semiconductor forms the diffusion region of the source and drain, which five contact surfaces can define. These surfaces form PN junctions created by the drain (source) region, which is in planar contact with the surrounding type-p and p+ semiconductors. In Figure 4, we can see these five surfaces, labelled from 1 to 5. Each of these parasitic capacitances is calculated separately according to the specific PN junctions, and the resulting capacitance of the entire diffusion region of the drain (source) is given by the sum of these capacitances. For simplicity, we assume that the n+ region is a cuboid with dimensions: width *W*, length *Y*, and thickness $X_{j}$. We assume that none of these five areas has the same PN junction. Areas 1 and 5 are formed by a PN junction of type n+ and p (n+ <-> p), and areas 2, 3, and 4 are formed by junction n+ and p+ (n+ <-> p+).

In practice, the actual shape of the area is quite complex and the concentration of impurities is not uniform. Since the voltage at these PN junctions also changes dynamically during the operation of the transistor, it is quite complicated to determine the exact number and value of these capacitances. The junction capacitances at zero bias voltage of the PN junction are determined by the capacitance $C_{j}$, multiplied by the area parameters AD and AS, and by the capacitance $C_{\mathrm{jsw}}$, multiplied by the area parameters PD and PS. The absolute values of $C_{\mathrm{DB}}$ and $C_{\mathrm{SB}}$ can also determine these junction capacitances. The bottom-plate capacitance, associated with the bottom of the junction, $C_{j}$ is the elementary junction capacitance given per unit area, and the parameters AD and AS are the drain and source diffusion areas, respectively, determined by area sizes 1 and 5 (Figure 4). The sidewall capacitance due to the perimeter of the junction, $C_{\mathrm{jsw}}$ is the elementary junction capacitance given per unit length. We assume that the thickness of the source and drain diffusion regions are the same around the entire perimeter. The parameters PD and PS are the drain and source diffusion perimeter respectively, determined from surfaces 2, 3, and 4 shown in Figure 4. The total capacitance at zero bias of the PN junction is given by the sum of the capacitances of all the surfaces shown in Figure 4. Since we assume that the substrate is connected to the ground, the PN junction’s bias voltage depends on the voltage $V_{\mathrm{DS}}$, which creates a voltage $V_{\mathrm{DB}}$ on the PN junction and polarizes the PN junction in reverse bias. The total capacitance calculation also depends on the voltage potential $\psi_{0}$ of the PN junction (see Table 1). The $C_{\mathrm{DB}0}$ capacitance is the total capacitance given by the sum of the capacitances of region areas 1 to 5 at zero bias voltage of the PN junction. The total capacitance of the diffusion region of the drain or source as a function of voltage is given by the relation [23]:

(8) $$C_{\mathrm{DB}}=\frac{C_{\mathrm{DB}0}}{\sqrt{1+V_{\mathrm{DB}}/\psi_{0}}}$$

(9) $$C_{\mathrm{SB}}=\frac{C_{\mathrm{SB}0}}{\sqrt{1+V_{\mathrm{SB}}/\psi_{0}}}.$$

However, simple reasoning shows that the resulting size of these capacities is most dependent on the region area of the PN junction. Based on the knowledge of these capacitance effects, the gate capacitance effect and the capacitance effect formed at the PN junction of the diffusion regions of the drain (source), we can conclude that by reducing the dimensions of the conductive channel, we can reduce the gate capacitance effect. By reducing the dimensions of the diffusion regions of the drain and source, we can reduce the value of the capacitances between the drain (source) and the body.

## 3. Circuit Analysis

### 3.1. Analysis and Functional Description of the Switch

This section describes the principle of operation of the high-frequency switch. The switch type was selected based on the requirements defined for the circuit design presented in Section 4. From the described theoretical knowledge about switches and different types of topological circuits, the topology of the series-parallel switch satisfied these requirements the most. From the description of the operation of the actual switching element used in this switch design, we can analyze the behavior of the whole switch in different functional states. The described characteristics and behavior of the switch described in this paper assume the implementation of the switch in 0.35 µm SiGe BiCMOS technology. These switch properties and behavior are only in principle for other fabrication technologies.

**Figure 5:**
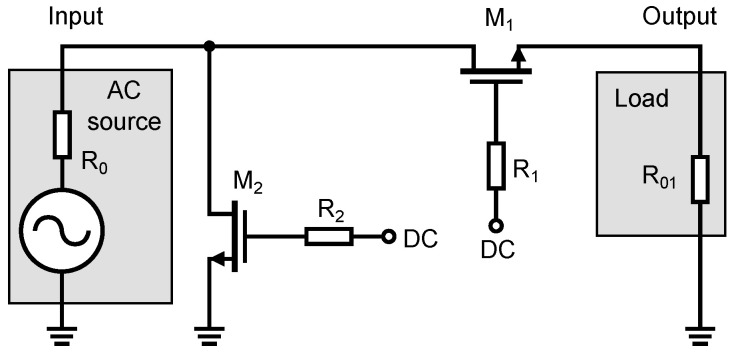
Basic circuit diagram of the high-frequency switch.

**Figure 6:**
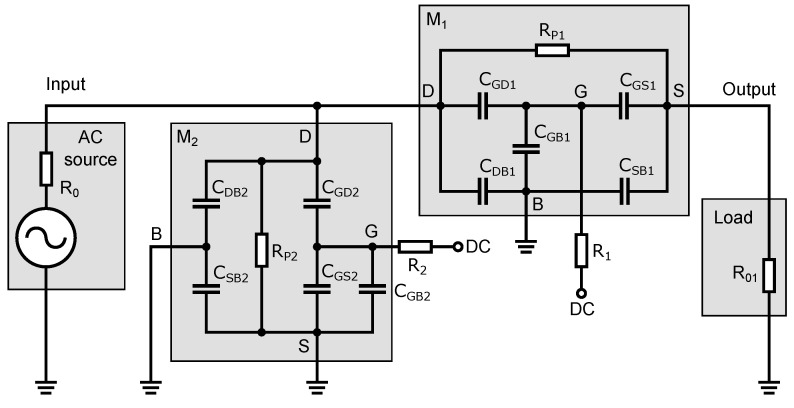
Equivalent circuit diagram of the switch with the NMOS transistor capacitance model.

Figure 5 shows the basic circuit diagram of a high-frequency switch. The circuit consists of two NMOS transistors, an input signal source and a load. The high-frequency signal source connected to the input generates a signal that is either disconnected or connected to the output of the load, depending on the DC voltages applied to the gates of transistors $M_{1}$ and $M_{2}$. The values of this DC control voltage depend on the type and the threshold voltage of the NMOS transistor. Resistor $R_{0}$ in the signal source and resistor $R_{01}$, forming the load, are essential for the optimal operation of the switch. These resistors ensure the impedance matching of the high-frequency signal on the input and output. For the UWB systems, the impedance matching value is typically 50 Ω. Resistors $R_{0}$ and $R_{01}$ are the same value. After analyzing the transistor in the different operating modes and assuming its application in the RF range and hence the associated existence of parasitic capacitances in the NMOS transistor structure, an equivalent circuit diagram has been developed based on the parasitic capacitance in a MOS transistor of Figure 2. The equivalent schematic shows all parasitic capacitances as well as resistive elements that are substitutes for the transistors, independent of the turn-on/off state of the transistors. The equivalent schematic is shown in Figure 6.

**Figure 7:**
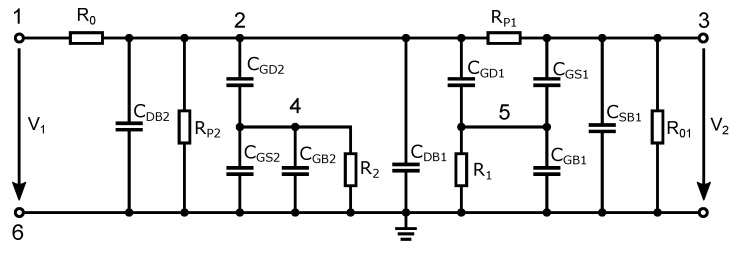
Small-signal equivalent circuit of the switch.

As shown in Figure 6, the transistor replacement circuit consists of five capacitances, $C_{\mathrm{GS}}$, $C_{\mathrm{GD}}$, $C_{\mathrm{GB}}$, $C_{\mathrm{SB}}$, and $C_{\mathrm{DB}}$, and a switching resistance $R_{p}$. All elements’ values vary depending on the turn-on/off state of the transistor and the amplitude of the applied signal from the high-frequency source. To analyze the behavior of the signal that passes through the switch, a small-signal equivalent circuit of the switch (Figure 7) was constructed from an equivalent circuit diagram (Figure 6). In this schematic; some circuit elements have been rearranged and neglected. The parasitic capacitance $C_{\mathrm{SB}}$ of the transistor $M_{2}$ is connected between the ground terminals of an equivalent circuit diagram in Figure 6. Thus, it is shorted out and, therefore, it does not affect the signal transmission through the switch and can be omitted.

We assume that the DC voltage source behaves as a short to ground for the high-frequency signal, therefore resistors $R_{1}$ and $R_{2}$, which are used to decouple the gate capacitances of $C_{\mathrm{GS}}$, $C_{\mathrm{GD}}$, and $C_{\mathrm{GB}}$, are connected to ground in the in the small-signal equivalent circuit of the switch. To identify the parasitic capacitances and resistive elements to the individual transistors, these elements are denoted by index 1 and 2 (e.g., $C_{\mathrm{GD}1}$). This notation is used because the parasitic capacitances are different depending on the dimensions of the transistor as well as on the different operating modes of the transistor, i.e., with the switch on or off. This indexing will be used when analyzing the individual switch states and the circuit simulation in Matlab.

We will use the nodal voltage analysis to calculate the signal transfer, on the basis of which we can construct the admittance matrix $Y^{*}$ (Equation (11)). In the schematic in Figure 7, we have labeled the nodes with the numerical values 1 to 6. The voltage transfer of the switch can be calculated according to the following relation:

(10) $$A_{u}=S_{21}=\frac{V_{36}}{V_{16}/2}=\frac{2\Delta_{16:36}^{*}}{\Delta_{16:16}^{*}}.$$

Then, we create the resulting admittance matrix $Y^{*}$ which, according to the number of nodes, will contain 6 rows and 6 columns. Equation (10) shows that rows 1 and 6 and column 6 are unnecessary for the calculation and can therefore be omitted. The notation $\Delta_{16:36}^{*}$ represents the algebraic complement of the matrix $Y^{*}$, which has rows 1 and 6 and columns 3 and 6 omitted. The algebraic complement $\Delta_{16:16}^{*}$ of matrix $Y^{*}$ has rows 1 and 6 and columns 1 and 6 omitted. In impedance matching, we divide the internal voltage of the signal source by two because, at the output terminal of the source, we assume that half of the voltage drop remains on the internal resistor of the signal source and half of the voltage drop remains on the input resistor of the switch.

(11) $$Y_{16:6}^{*}=\left(\begin{array}{ccccc} -G_{0} & \begin{array}{c} G_{0}+G_{p}+G_{p2}\\ +pC_{\mathrm{DB}}+pC_{\mathrm{GD}}\\ +pC_{\mathrm{DB}}+pC_{\mathrm{GD}} \end{array}\; & -G_{p} & -pC_{\mathrm{GD}} & -pC_{\mathrm{DB}}\\ 0 & -G_{p} & \begin{array}{c} G_{0}+G_{01}\\ +pC_{\mathrm{SB}}+pC_{\mathrm{GS}} \end{array}\; & 0 & -pC_{\mathrm{GS}}\\ 0 & -pC_{\mathrm{GD}} & 0 & \begin{array}{c} G_{2}+pC_{\mathrm{GD}}\\ +pC_{\mathrm{GS}}+pC_{\mathrm{GB}} \end{array}\; & 0\\ 0 & -pC_{\mathrm{GD}} & -pC_{\mathrm{GS}} & 0 & \begin{array}{c} G_{1}+pC_{\mathrm{GD}}\\ +pC_{\mathrm{GS}}+pC_{\mathrm{GB}} \end{array} \end{array}\right).$$

**Figure 8:**
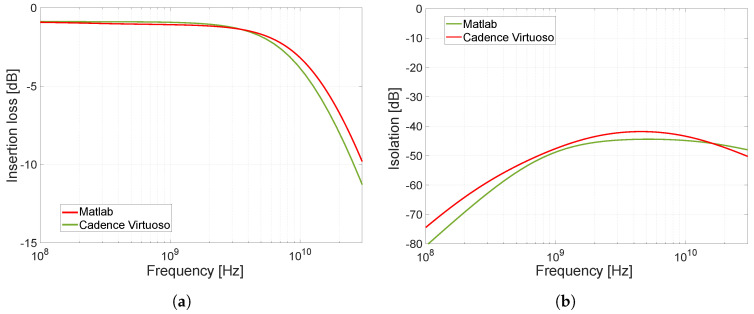
Comparison of results obtained from Matlab and Cadence CAD design tool. (**a**) Comparison of calculated insertion loss. (**b**) Comparison of calculated isolation.

The previous analysis of the switch equivalent circuit diagrams in different modes of operation evaluated the influence of electronic components and parasitic elements on its operation and functionality. Since this analysis is the basis for the design of our precisely designed and optimized switch, the correctness of the conclusions introduced was also verified analytically based on the admittance matrix. The admittance matrix was derived from the equivalent circuit diagrams presented in the sections dealing with analysis and, as already mentioned above, also takes into account parasitic elements affecting the required properties of the proposed switch. From the size of the matrix and the number of elements, it is clear that the manual computation of the algebraic complements, the transfer function, and the subsequent depiction of the transfer function are very complex and difficult. Because of this difficulty and the potential mistakes of manual calculation, a program was created in the Matlab environment to show the insertion loss and isolation. Thus, the insertion loss and isolation characteristics were computed from the admittance matrix in Matlab and these results were compared with those found from the simulations in the Cadence CAD design tool. This comparison of the results (see Figure 8) clearly confirms that our analysis and the models presented in the paper are in good agreement with the models used in the Cadence CAD design tool (specifically, the low-cost 0.35 µm SiGe BiCMOS technology was chosen). In the next sections, based on the designed models and the analysis of the influence of electronic components and parasitic elements of the model, the structure of the proposed switch will be optimized to meet the required parameters for our UWB applications.

### 3.2. The Closed State of the Switch

**Figure 9:**
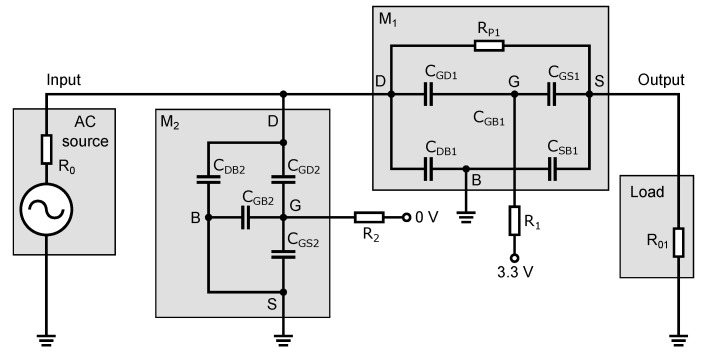
Schematic circuit diagram of the switch operating in the closed state with equivalent elements of the transistor model.

The behavior of the high-frequency switch in terms of high-frequency signals in the closed state is evident from Figure 9. In the closed state, transistor $M_{1}$ is open and a DC voltage of 3.3 V is applied to its gate. Transistor $M_{2}$ is closed and a zero voltage is applied to its gate. Transistor $M_{1}$ behaves as a resistive element with resistor $R_{p1}$. If the value of $R_{1}$ is too small, the RF signal will pass through the gate of transistor $M_{1}$, i.e., through the parasitic capacitances $C_{\mathrm{GD}1}$ and $C_{\mathrm{GS}1}$, to the DC control source, which results in the signal short to the ground and increasing the attenuation at high frequencies. The parasitic capacitances $C_{\mathrm{GD}1}$ and $C_{\mathrm{GS}1}$ can be calculated for the open state of the transistor $M_{1}$, according to Equation (6), by adding the value of the overlapping capacitances $C_{\mathrm{GDs}}$ and $C_{\mathrm{GSs}}$ (see Table 1). The capacitance of $C_{\mathrm{GB}1}$ is zero for the open state of the transistor $M_{1}$ and can be omitted.

The appropriate value of resistor $R_{1}$ eliminates the effect of capacitances $C_{\mathrm{GD}1}$ and $C_{\mathrm{GS}1}$ on the switch’s turned-on state attenuation. Thus, of the parasitic capacitances of transistor $M_{1}$, only the junction capacitances $C_{\mathrm{DB}1}$ and $C_{\mathrm{SB}1}$ will have the greatest effect on the high-frequency signal.

Transistor $M_{2}$ is closed and its channel resistance approaches infinity. This means that transistor $M_{2}$ is capacitive in nature and its parasitic capacitances also affect the transmission of the high-frequency signal. Since transistor $M_{2}$ is closed, the capacitance of $C_{\mathrm{GB}2}$ has a maximum value determined by Equation (5). Another capacitance affecting signal transmission is the junction capacitance of $C_{\mathrm{DB}2}$, which has a direct coupling to the ground. The parasitic capacitances $C_{\mathrm{GD}2}$ and $C_{\mathrm{GS}2}$ of the closed transistor $M_{2}$ have only the values of the overlap capacitances mentioned in Section 2.2. The values of these overlap capacitances are ten to twenty times lower than the value of the capacitance $C_{\mathrm{DB}2}$ and so the capacitance $C_{\mathrm{GD}2}$, through which part of the RF signal passes to resistor $R_{2}$ and the capacitances $C_{\mathrm{GB}2}$ and $C_{\mathrm{GS}2}$, does not have a large effect on the output RF signal. Even if the capacitances $C_{\mathrm{GB}2}$ and $C_{\mathrm{GS}2}$ have a higher value than the capacitance of $C_{\mathrm{GD}2}$ by simply considering a series-parallel connection of the capacitances of $C_{\mathrm{GD}2}$, $C_{\mathrm{GS}2}$, and $C_{\mathrm{GB}2}$, we can conclude that the total value of the parasitic capacitance that drives the signal to the ground will be slightly less than the capacitance $C_{\mathrm{GD}2}$. This means that removing the resistor $R_{2}$ and shorting the capacitances $C_{\mathrm{GB}2}$ and $C_{\mathrm{GS}2}$ has a negligible effect on transmitting the high-frequency signal through the switch. For the limiting case where the signal frequency approaches zero, or equivalently for low-frequency signals not yet affected by parasitic capacitances, we can simply determine the signal transmission by the equation:

(12) $$A_{v}=S_{21}=\frac{V_{36}}{V_{16}/2}=\frac{2V_{2}}{V_{1}}=\frac{2R_{0}}{2R_{0}+R_{p}}=\frac{R_{0}}{R_{0}+R_{p/2}}\;if:R_{01}=R_{0}.$$

### 3.3. The Open State of the Switch

**Figure 10:**
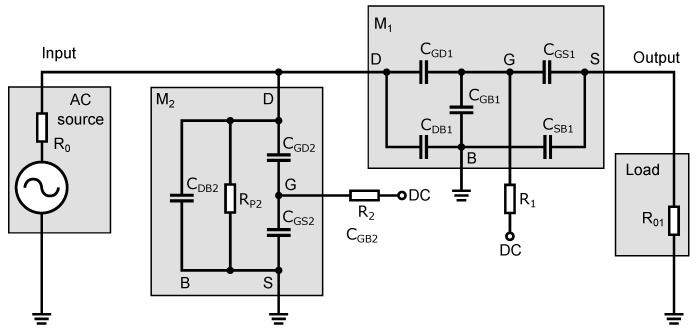
Schematic circuit diagram of the switch operating in the open state with equivalent elements of the transistor model.

The operation and behavior of the switch in the open state are evident from the diagram shown in Figure 10. In the open state, transistor $M_{1}$ is closed; zero voltage is applied to its gate. Transistor $M_{2}$ is open through a DC voltage of 3.3 V applied to the gate of the transistor. Transistors $M_{1}$ and $M_{2}$ behave oppositely to the previous case. The resistance of transistor $M_{1}$ in the open state has a very high value, and the transistor is only capacitive in nature with capacitances $C_{\mathrm{DB}1}$, $C_{\mathrm{GD}1}$, $C_{\mathrm{GB}1}$, $C_{\mathrm{GS}1}$ and $C_{\mathrm{SB}1}$ through which the signal passes both to the output and to ground. While in the closed state of the switch, the value of resistor $R_{1}$ has been set so that the capacitances $C_{\mathrm{GD}1}$ and $C_{\mathrm{GS}1}$ do not adversely affect the frequency bandwidth and the value of the attenuation, in the open state of the switch, this resistor prevents these capacitances from coupling to ground, causing the signal to be transmitted through the closed transistor to the output and reducing the isolation. This is partially compensated for by the capacitance $C_{\mathrm{GB}1}$, which has a maximum value determined by Equation (5). The parasitic capacitances $C_{\mathrm{GD}1}$ and $C_{\mathrm{GS}1}$ of the closed transistor $M_{1}$ have only the values of the overlap capacitances.

Transistor $M_{2}$ is open and behaves as a resistive element. This transistor’s role in the open state of the switch is to increase the isolation of the switch. In the case when the transistor $M_{2}$ is open, the capacitance $C_{\mathrm{GB}2}$ has a zero value and the capacitances $C_{\mathrm{GD}2}$ and $C_{\mathrm{GS}2}$ have values that can be calculated according to Equation (6) with the addition of the values of the overlapping capacitances $C_{\mathrm{GDs}}$ and $C_{\mathrm{GSs}}$ (see Table 1). With higher capacitance, these gate capacitances have a greater effect on the RF signal than it was in the case of the closed transistor $M_{2}$. The capacitance $C_{\mathrm{DB}2}$ is determined by the junction area’s size in the drain’s diffusion region. In the previous subsection, we stated that resistor $R_{2}$, due to the small capacitance of $C_{\mathrm{GD}2}$, has a minimal effect on the RF signal transmission because transistor $M_{2}$ was closed. When increasing the capacitances $C_{\mathrm{GD}2}$ and $C_{\mathrm{GS}2}$ of the open transistor $M_{2}$, a non-zero resistance value of resistor $R_{2}$ would have a negative effect on the isolation. This resistor has a negative effect because the capacitances $C_{\mathrm{GD}2}$ and $C_{\mathrm{GS}2}$ form a series connection (see Figure 10), and a series connection at the same capacitance values implies that the resulting capacitance will be half, reducing the isolation. By eliminating resistor $R_{2}$, we short-circuit the capacitance $C_{\mathrm{GS}2}$, and only the capacitance $C_{\mathrm{GD}2}$ will affect the HF signal pull-down to the ground. Therefore, this resistor has been omitted in implementing the switch design.

## 4. Design of the Integrated UWB Switch

The design of the switch is implemented in BiCMOS technology and was based on the theoretical knowledge described in the previous chapters and on simulations carried out in a Cadence CAD design tool with the basic electronics components models of the 0.35 µm SiGe BiCMOS technology used. Before starting the circuit design of a broadband integrated semiconductor switch, it is necessary to establish the characteristics the designed circuit should satisfy:

- Minimum, i.e., as small signal attenuation as possible in the closed state (ideally 0 dB);
- Maximum on-chip isolation in the open state, preferably below −40 dB;
- A switch frequency bandwidth as wide as possible, from the lowest radio frequencies to a minimum of 10 GHz;
- The highest possible power that the switch can transmit without compression/distortion;
- A control voltage of 3.3 V for logic and switching needs;
- Low power consumption;
- A define the number of outputs, in our case three outputs for the UWB sensor systems (e.g., when switching and configuring an antenna MIMO system) or for switching calibration tools (primarily intended for sensor system calibration).

Based on the above requirements, the high-frequency part of the switch consists of three identical series-parallel switches. Since the switches are designed, e.g., for the calibration of RF devices, the individual outputs can be connected to the components used in the device calibration. The control logic specifies the terminal, e.g., with the corresponding calibration element. A 50 Ω load, a short circuit, and an open output are used for calibration. A detailed schematic will be given and its operation will be explained in the next section.

### Design and Simulation of the High-Frequency Part of the Switch

**Figure 11:**
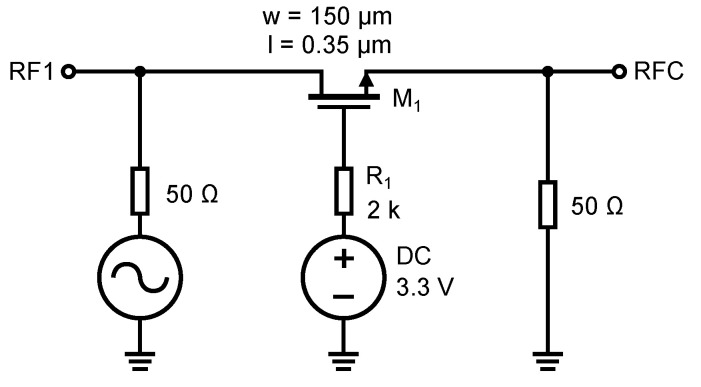
Schematic circuit diagram of the switch to simulate the effect of the width of the transistor $M_{1}$ and the resistor $R_{1}$.

**Figure 12:**
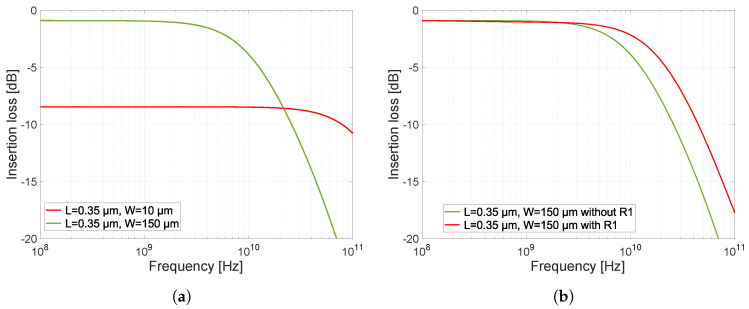
Simulations of series switch with one NMOS transistor. (**a**) Characteristics of the effect of $M_{1}$ transistor width on insertion loss and frequency bandwidth. (**b**) Characteristics of the influence of the presence of resistance $R_{1}$ on the frequency bandwidth of the switch.

It should be noted at this point that the design and simulations do not consider the parasitic properties of the bonding pads and conductive paths on the PCB upon which the switch could be designed and implemented for a particular UWB application. However, their influence will be considered and shown through the measurement results presented in Section 6. The first design and simulation optimized the signal transmission through the switch in the turned-on state. The simulation was performed on a simple serial switch with a single NMOS transistor. The schematic is shown in Figure 11. A switching voltage of 3.3 V is applied to the gate of the transistor, which opens transistor $M_{1}$. The transmission parameter S21 (S12) through the switch depends largely on the geometrical dimensions of the transistor, length *L* and width *W*. We keep the length *L* as low as possible because increasing the length increases the conduction channel resistance and parasitic capacitance. Increasing the transistor’s width decreases the open transistor’s resistivity and reduces the signal attenuation. The simulations of the characteristics in Figure 12 were performed without resistor $R_{1}$.

However, increasing the channel width has an undesirable effect on the frequency bandwidth of the switch. As a result of increasing the channel width, the capacitances $C_{\mathrm{DB}}$, $C_{\mathrm{SB}}$, $C_{\mathrm{GD}}$, and $C_{\mathrm{GS}}$ become larger and start to affect the high-frequency signal more, resulting in a decrease in the frequency bandwidth of the switch. The $C_{\mathrm{DB}}$ capacitance does not exist when the transistor is open (see Section 3.2). The effect of the size of the transistor on its characteristics can be seen from the characteristics in Figure 12. In the simulation, the transistors with two different sizes were compared. Both transistors have a channel length equal to *L* = 0.35 µm, but the channel width differs. One has a *W* = 150 µm width, and the other has a *W* = 10 µm width. It can be seen that with the smaller transistor, the frequency bandwidth is much larger, but the attenuation is at about −8.6 dB. Conversely, with the wider transistor, the frequency bandwidth is smaller but the attenuation drops only to −1 dB. As a result, there is a trade-off to be made between attenuation and frequency bandwidth. The resulting dimension of transistor $M_{1}$ was chosen L×W= 0.35 × 150 µm.

The parasitic gate capacitances $C_{\mathrm{GS}1}$ and $C_{\mathrm{GD}1}$ feed a signal directly to the ground via a DC voltage source. Since the transistor is open, these capacitances are larger and, for high signal frequencies, these capacitances start to present a minimal impedance. The value of these capacitances is defined by Equation (6). To remove the undesirable influence of the parasitic capacitances $C_{\mathrm{GS}1}$ and $C_{\mathrm{GD}1}$, we connect resistor $R_{1}$ to decouple the gate potential from the DC source. By adding resistor $R_{1}$, we increase the impedance created by the $C_{\mathrm{GD}}$ and $C_{\mathrm{GS}}$ capacitances, which decreases as the signal frequency increases. NMOS transistors have an insulated gate (by a thin layer of silicon dioxide, SiO_2_) and, since no current flows into the gate even after adding a resistor, the control voltage for the transistor is not reduced by the voltage drop across this resistor. The effect of the resistor has been simulated according to the schematic in Figure 11, and the resulting characteristic is shown in Figure 12b.

The characteristic shows the difference in bandwidth without resistor $R_{1}$ and after its connection to the gate of the transistor $M_{1}$. The resulting value of resistance of resistor $R_{1}$ was determined by simulation, and its value is 2 kΩ. Further increasing the resistance did not affect the increase in frequency bandwidth anymore. This is because we isolated by the resistor $R_{1}$ the parasitic capacitances to such an extent that the bandwidth was only affected by the junction capacitances $C_{\mathrm{DB}1}$ and $C_{\mathrm{SB}1}$ anymore.

By determining the dimensions of transistor $M_{1}$ and the value of resistor $R_{1}$, we set the frequency bandwidth and the attenuation in the switch’s closed state. The value of the resistance of $R_{1}$ contributed to the capacitances $C_{\mathrm{GS}1}$ and $C_{\mathrm{GD}1}$ no longer being tied to the ground. The schematic in Figure 9 shows that they are connected in series with the input and output terminal and thus affect the isolation in the open state of the switch. Although the values of these capacitances are smaller in size in the closed state of transistor $M_{1}$ than in the open state, they still represent a conducting path for the high-frequency signal.

**Figure 13:**
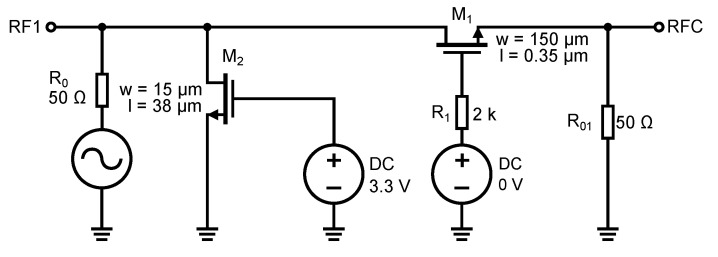
Schematic circuit diagram of the switch to simulate the effect of $M_{2}$ transistor dimensions on attenuation, frequency bandwidth, and switch isolation.

**Figure 14:**
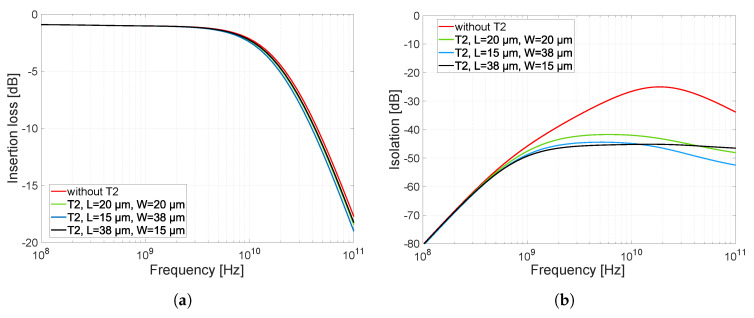
Effect of different dimensions of transistor $M_{2}$ on the value of attenuation, frequency bandwidth, and isolation. Dimensions are in the form of L×W, and the values of these dimensions are in µm. (**a**) Insertion loss, (**b**) isolation.

To increase the isolation in the closed state of the switch, transistor $M_{2}$ was added in parallel to drive the signal to the ground. The circuit diagram on which the simulations were performed is shown in Figure 13. Transistor $M_{2}$ must be set to minimize the attenuation and frequency bandwidth in the closed state of the switch and maximize the isolation in the open state. Since we analyzed in Section 3.2 and Section 3.3 that the presence of resistor $R_{2}$ has a minimal effect on the attenuation and frequency bandwidth in the closed state and only a small beneficial effect on the isolation in the open state (as confirmed by simulations), this resistor was omitted from the scheme. When resistor $R_{2}$ is removed, transistor $M_{2}$ acts in the switch circuit only as parasitic capacitances $C_{\mathrm{DB}2}$ and $C_{\mathrm{GD}2}$. Figure 14 shows the attenuation (Figure 14a) and isolation (Figure 14b) characteristics as a function of the dimensions of transistor $M_{2}$. The results show that a longer and narrower transistor is better than a wider and shorter one, the opposite of what was the case for transistor $M_{1}$. This is because, in the closed state of the switch, the attenuation and bandwidth are most affected by the capacitance of $C_{\mathrm{DB}2}$. The capacitances of $C_{\mathrm{DB}2}$ and $C_{\mathrm{GD}2}$ depend on the transistor’s width.

The width of the transistor directly changes the size of the junction areas of the source and drain regions (see Figure 4). The sizes of the junction areas retain their *Y* dimension, independent of the transistor length. The capacitance $C_{\mathrm{GD}2}$, when the transistor $M_{2}$ is closed, has a very low effect on the attenuation and frequency bandwidth since its value is minimal and is equal only to the value of the overlap capacitances. On the contrary, in the open state of the switch, the state of transistor $M_{2}$ changes; it opens. The capacitance of $C_{\mathrm{DB}2}$ will have the same value because it is independent of the $V_{\mathrm{GS}}$ voltage. In the open state of switch, transistor $M_{2}$ affects the isolation by its conducting channel and the capacitances $C_{\mathrm{GD}2}$ and $C_{\mathrm{DB}2}$. Increasing the width of the transistor $M_{2}$ has a negative effect on the signal in the closed state of the switch. On the other hand, increasing the transistor length has a beneficial effect on the isolation and negligibly affects the attenuation and frequency bandwidth. Due to the effect of the open transistor $M_{2}$ in the open switch state, the value of the capacitance $C_{\mathrm{GD}2}$ is much larger, and its value is increased by the effect of the larger transistor length. The calculation of the capacitance $C_{\mathrm{GD}2}$ is given by Equation (6). In the characteristics in Figure 14, this phenomenon, coupled with the length of transistor $M_{2}$, is visible. Based on the above, the resulting dimension of transistor $M_{2}$ was chosen as L×W = 38 × 15 µm. Transistor $M_{2}$ with these dimensions has good characteristics in terms of switch isolation, where the isolation value dropped below −45 dB.

**Figure 15:**
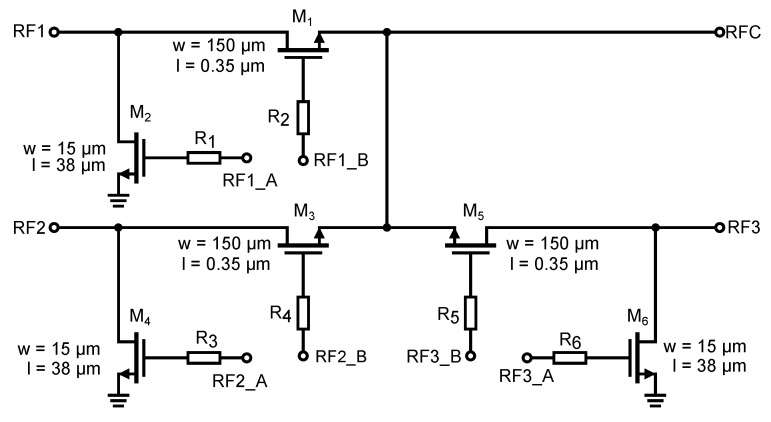
Overall circuit diagram of the implemented controlled semiconductor UWB switch.

**Figure 16:**
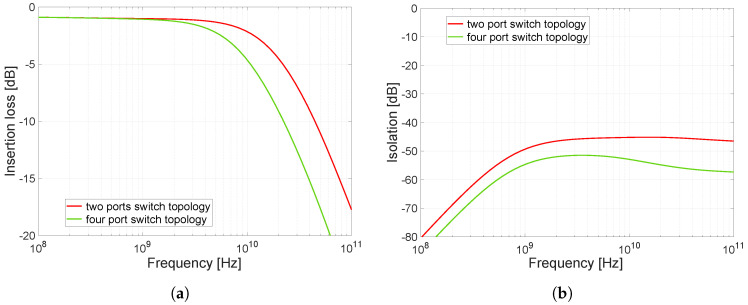
Comparison of one simple switch and three identical switches connected to one output. (**a**) Comparsion of insertion loss. (**b**) Comparison of isolation.

The design requirements for the resulting high-frequency switch indicated that more than one output was needed. Since three outputs, 50 Ω, short, and open-circuit, are primarily required for the applications under consideration, e.g., for calibration, the resulting circuitry of the proposed switch consists of three series-parallel switches (see Figure 15). One port of these switches is connected to a common RFC terminal and the other switch ports are determined by each switch separately. We have labelled the ports RF1, RF2, and RF3 for easy identification. The resulting circuitry of the entire high-frequency part of implemented integrated switch is shown in Figure 15. For the resulting circuitry of three identical switches with one output, the original transmission characteristics will change due to the mutual interaction of these individual switches. For the scheme shown in Figure 15, simulations were performed to determine the changes caused by this circuitry. The result of the simulation is shown in Figure 16.

It can be seen in the characteristics that there is a rapid decrease frequency bandwidth (Figure 16a). Conversely, it can be seen that there was a beneficial effect in the isolation area (Figure 16b) and the isolation dropped to below 53 dB, which exceeds the desired isolation requirement. The frequency bandwidth has decreased to approximately 8 GHz (note that the bandwidth intervals in this paper are given at a −3 dB drop; however, in some papers discussing UWB, we may encounter bandwidth intervals given at a 10 dB drop).

However, the resulting parameters will still change after the layout design. The change in characteristics is justified in the capacity $C_{\mathrm{SB}1}$ (see schematic in Figure 6 or Figure 7). The mutual coupling of the three switches means that the capacitances from transistors $M_{1}$, $M_{3}$ and $M_{5}$ are connected in parallel in the resulting schematic, resulting in a reduction in frequency bandwidth in the closed state and an increase in isolation in the open state of the switch.

From the previous simulations and setups, we determined the resulting transistor dimensions. Transistors $M_{1}$, $M_{3}$, and $M_{5}$ connected in series have a resulting dimension of L×W = 0.35×150 µm, and transistors $M_{2}$, $M_{4}$ and $M_{6}$ connected in parallel have a resulting dimension of L×W= 15×38 µm.

## 5. Implementation of the Integrated UWB Switch

**Figure 17:**
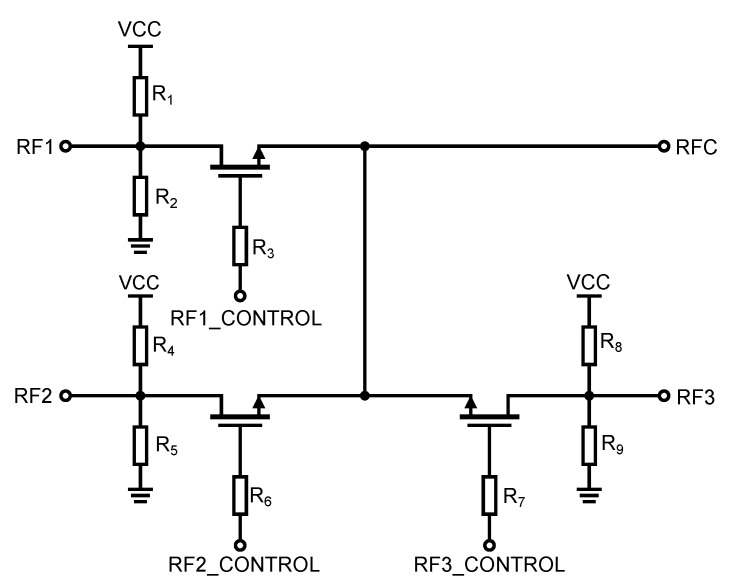
Schematic of the implemented integrated UWB switch.

Today’s ASIC design implementation flow must address different design modes, such as functional modes, test modes, and several input/output functions at different process, voltage, and temperature (PVT) corners, as its main function. Based on the analysis of the RF switch and NMOS transistor [20], a designed UWB switch was on-chip implemented as an application specific integrated circuit (ASIC) with additional tested structures for UWB applications. The resulting circuit diagram of the implemented integrated UWB switch is shown in Figure 17. For the sake of simplicity and ease of testing, the original schematic presented in the analysis (Figure 15) was simplified to a common SP3T (Single Pole Three Throw) switch without transistors $M_{2}$, $M_{4}$, and $M_{6}$. Additionally, the resistors were added to the resulting schematic to set the internal DC bias to 1.65 V, half the $V_{\mathrm{gs}}$ voltage. The original analysis was performed for the switch behavior at a standard 3.3 V power supply, where setting $V_{\mathrm{gs}}$ = 3.3 V resulted in the switch being on or in the closed state. For compatibility with the UWB transceiver [13], it was simpler to use a power supply of −3.3 V for the switch, when by applying the voltage $V_{g}$ = 0 V (GND potential), a potential of $V_{\mathrm{gs}}$ = 3.3 V is again created, which closes the switch.

The problem occurs when the AC signal is connected, which has a DC value of 0 V, i.e., the potential of GND. In this case, the switch will not be closed by applying $V_{g}$ = 0 V because the voltage difference $V_{\mathrm{gs}}$ = 0 V. Therefore, DC bias adjustment resistors have been added to the final implementation so that the switch can be opened even using $V_{g}$ = 0 V. This results in a voltage drop $V_{\mathrm{gs}}$ = 1.65 V, which is sufficient to open the NMOS transistor.

Before the layout of the proposed and implemented UWB switch, it is necessary to be sure that the proposed design is working properly. Thus, in the next steps, we will be going from a schematic to the final layout (tape out). The functionality of the design at different levels was verified and the performance, timing, and power dissipation parameters important for our design were simulated. The most advanced IC technologies available to us and Cadence’s professional CAD tools have been used. Specifically, we followed the next steps in our practical ASIC design implementation:

- Schematic Design—A schematic based design flow and simulation were used. A typical ASIC design implementation includes schematics (consisting of transistor-level Specification & Functional verification) for functional mode as well as the specified technology library. Upon completion of the design schematic, the final netlist (pre-layout netlist) to the physical design for place and route was provided.
- Physical Design—Manual placement and drawing of all devices and structures were used. Once the pre-layout netlist is available, the physical design activities will begin. During the physical design activities, the goal will be to first make sure the routed design will meet the physical design rules (Design Rule Check) and then, if both the schematic circuit and layout circuit match. Upon completion of the routed design, the physical design will provide the post-layout netlist for the final optimization, verification, and evaluation. This uses the simulation same as the simulation of the schematic except for the fact that the results will now include parasitic effects from the actual layout (Extraction). Parasitic capacitances, resistances, and inductances in the layout can strongly affect the performance of a design. Thus to evaluate the effects of parasitics and to gain a higher degree of confidence that a layout will result in a chip that meets the specifications, it is important to run post-layout simulations, as well. In our design flow, we only extracted a layout with parasitic capacitors and resistors (our design environment also supports the extraction of RLC parasitics, but parasitic inductors are not included in the design kit) and only with their values above a certain chosen limit level so that their influence does not significantly affect the operation or accuracy of our design. The performance of a circuit simulated with parasitics accounted for is always worse, although closer to reality, than a schematic that does not include estimated parasitics. Thus, this analysis examines the operation of the design across a range of processes and also different conditions (like voltage, temperature, crosstalk etc.).

Once we are done, we will have a prototype layout for our ASIC UWB switch that can be sent for fabrication (tape out, GDS II). The chip can only be sent for fabrication only if proof of functionality from post-layout simulations is provided.

**Figure 18:**
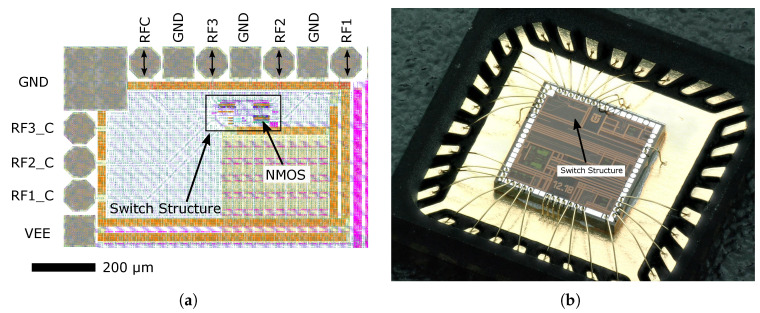
View of designed structures of the switch. (**a**) The layout of the proposed and implemented UWB switch. (**b**) Switch die mounted into a QFN open-top package.

Figure 18 shows the layout of the proposed and implemented switch structure, where the above-mentioned NMOS transistors with size L×W = 0.35×150 µm can also be seen. The high-frequency ports RFC, RF1, RF2 and RF3 are located on the top side of the switch, and the individual control inputs RF1_C- RF3_C for the gates of the NMOS transistors are located on the left side.

## 6. Measurement of the Integrated UWB Switch

**Figure 19:**
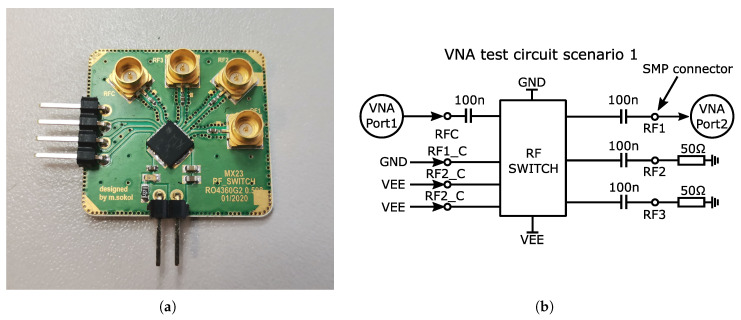
Packaged UWB switch. (**a**) Designed prototype of RF switch soldered to development board. (**b**) Block diagram schematic of mesurement scenario with VNA Agilent N5241A.

This section presents the measurement and test results of the fabricated integrated UWB switch. The realized UWB switch was wire-bonded in the package (die mounted into a QFN32 5 × 5 mm) and soldered on the custom testing a precision-designed printed circuit board (PCB), made of RO4360G2 substrate with dielectric constant ε_r_ = 6. The finished PCB prototype is shown in Figure 19a. The connection and measurement scenario of the prototype integrated UWB switch was the same as that of the simulations that were also performed. A Vector Network Analyser (VNA) Agilent N5241A PNA X (max 13.5 GHz) was used for the measurements. A block diagram of the test circuit scenario is shown in Figure 19b. Due to only two ports of the VNA device being available, only two ports of the switch were measured simultaneously, the other ports were terminated with a 50 Ω load. In order to internally decouple the DC bias voltage, 100 nF RF capacitors were added as DC blocks. The 100 nF capacitors provide AC coupling from tens of kHz.

**Figure 20:**
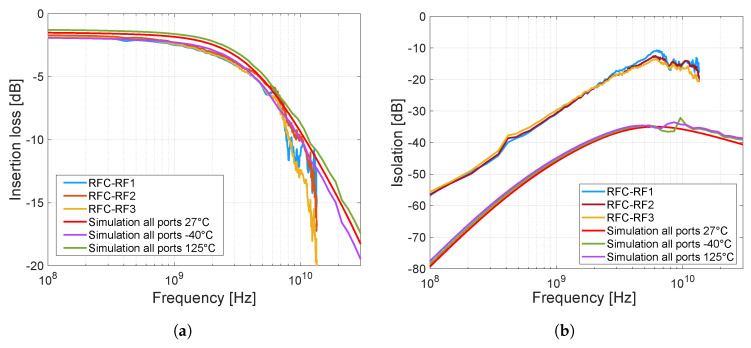
Insertion loss and isolation of individual ports of the switch obtained from measurements (100 MHz–13.5 GHz) and post-layout simulations (100 MHz–30 GHz). (**a**) Insertion Loss from RFC to other ports, sig.bias = internal, load = 50 Ω. (**b**) Isolation from RFC to other ports, sig.bias = internal.

The insertion loss and isolation of each port of the switch obtained from measurements and post-layout simulations are shown in Figure 20. Post-layout simulations were performed in frequency range from 100 MHz to 30 GHz, and in three temperature variations based on full-range industry standard −40 °C to 125 °C. The insertion loss was measured separately for three switching possibilities, i.e., between the RF1 port and the RFC port, RF2 – RFC, and RF3 – RFC, based on the scenario in Figure 19b. First, port RF1 was in the closed state and the others were turned to open, this was repeated for additional ports RF2 and RF3. The measurement of isolation was performed similarly, but the measured port was closed. By measuring the insertion loss of the fabricated switch, it can be seen (Figure 20a) that this insertion loss and frequency bandwidth are almost identical to the post-layout simulation of the proposed design. In contrast, the isolation measurements of the fabricated switch (Figure 20b) are already different from the post-layout simulations of the proposed design (by about −15 dB). The worse measured isolation result is due to higher crosstalk on the chip and on the PCB. This can be caused by several parameters such as the size of pads and the geometry of the lines on the chip.

**Figure 21:**
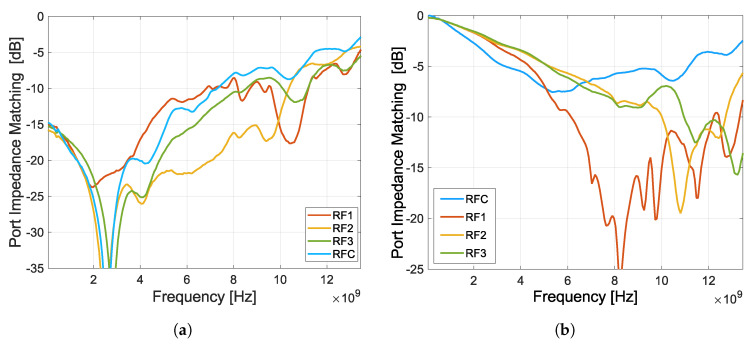
The 50 Ω port matching measurements for each port separately in closed and open state. (**a**) Switch port matching close state, VEE = −3.3 V, scenario 1. (**b**) Switch port matching open state, VEE = −3.3 V, scenario 1.

**Figure 22:**
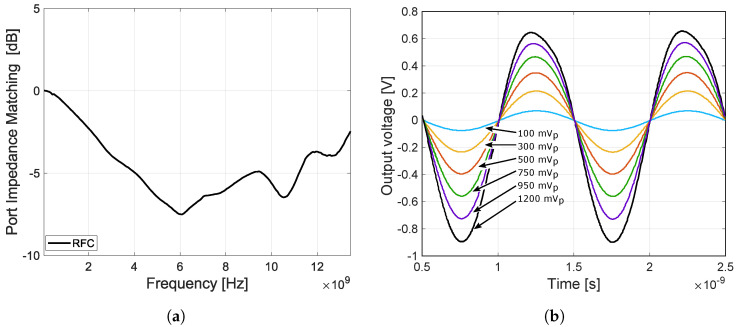
Measurements of RFC port matching ALL-OFF state and voltage swing. (**a**) RFC port matching all open state, VEE = −3.3 V, scenario 1. (**b**) Measurement of a voltage swing, Load = 50 Ω, input signal frequency = 1 GHz, input sig.bias = internal, output is AC coupled to load, VEE = −3.3 V, scenario 1.

The 50 Ω port matching measurements are shown in Figure 21. The matching was measured for each port separately in closed and open states. Due to the reflective topology of the designed switch, the UWB switch ports are not matched to the system impedance when the ports are open. A measurement of the RFC port matching was also performed for the case when all other ports were open. These measurement results are shown in Figure 22a.

The last measurement is shown in Figure 22b. For this measurement, a 1 GHz sinusoidal signal with amplitudes of 100, 300, 500, 750, 950, and 1200 mV_p_ was applied to port RF1. From this measurement, the attenuation and compression of the sinusoidal signal can be seen. The compression is caused by reducing the voltage difference $V_{\mathrm{gs}}$, as a result of which the NMOS transistor enters the cutoff region.

## 7. Conclusions

The aim of this paper was the design and implementation of a particularly low-cost SiGe BiCMOS technology, and the simulation as well as the realization and evaluation of the characteristics of the high-frequency semiconductor integrated switch. The analysis of the chosen solution using the theoretical knowledge of MOSFET transistors and 0.35 µm SiGe BiCMOS technology helped design the presented switch. The switch design was realized after an initial presentation of the switch issues, the fabrication technology used, and the theory associated with the MOSFET transistor models. The design was implemented and the predicted theoretical behavior of the switch was verified using simulations in a Cadence CAD design tool. Based on these simulations, the parameters of the final design of the UWB switch were improved by successively modifying the circuit parameters. Simulations were also performed on the layout of the designed chip, including parasitic elements due to the influence of the transistor placements and conductive paths on the chip, as well. The resulting post-layout simulations confirmed the predicted estimates in terms of bandwidth. The resulting design achieves bandwidths ranging from a “DC” signal up to nearly 6 GHz, with a considered −3 dB decrease. The insertion loss at low frequencies is 1.2 dB and the isolation has a minimum value of −18 dB. Power simulations were also performed on the layout. The switch achieves a 1 dB compression point at the power of around 5 dBm, which represents a power of about 2.5 mW. The designed switch performance parameters achieved are sufficient for our required switch application. The power consumption of the switch reaches the order of units of mW, thanks to the MOSFET transistors used, which are characterized by very low power consumption. There is still room for improvement in this design; the goal is to obtain the best possible parameters out of the available fabrication technology and the MOSFET transistors themselves. Although for our UWB applications, the available power and bandwidth are sufficient, we need to continue to look for ways to increase the power transmitted by the switch. Further improvements to the design consist of refining the layout created, increasing isolation, and reducing inductances and parasitic capacitances. The layout of the switch is designed so far as a block without the die pads for the bonding wires, so it would be useful to further analyze the influence of these parasitic properties, comparing them with the results that were obtained from measurements on an implemented UWB switch mounted on a development board. Its precise design took into account our applications for UWB sensor systems, namely for the switching and configuration of the UWB radar antenna MIMO system or the switching of the calibration tools, primarily intended for the calibration of the UWB sensor system (three outputs). The main achieved parameters of the designed switch compared to a commercial RF switch [29] and other designs [30] implemented in 0.35µm CMOS are shown in Table 2.

## Figures and Tables

**Table 1:** Table of typical values of parasitic capacitances and parameters in 0.35 µm SiGe BiCMOS fabrication technology.

Parameter	Symbol	Typical Values	Unit
n+junction depth	$X_{j}$	0.18–0.22	µm
GATE to NDIFF overlap capacitance	$C_{\mathrm{GSDov}}$	0.3–0.4	fF/µm
Typical length of LDD	$L_{D}$	0.05*L* to 0.1*L*	fF/µm
GATE to LDD overlap capacitance	$C_{\mathrm{GSDL}}$	0.1–0.15	fF/µm
Elementary junction capacitance	$C_{j}$	0.7–1	fF/µm^2^
Sidewall junction capacitance	$C_{\mathrm{jsw}}$	0.2–0.3	fF/µm
Junction potential	$\psi_{0}$	0.7	V

**Table 2:** Comparison of manufactured switch presented in this article with commercial switch PE42540 [29] and other work [30].

Parameter	PE42540 [29]	This Work	[30] *
Max. supply voltage	3.55 V VDD −3.6 V VSS	−4.5 V VEE	-
Nominal supply voltage	3.3 V VDD, 3.3 V VSS	−3.3 V VEE	3 V VDD
Nominal supply current	200 µA	2 mA	-
Bandwidth	10 Hz–8 GHz	DC–6 GHz (−3 dB)	DC–6 GHz
Insertion loss (1 Ghz)	−1 dB	−2.2 dB	−2.1 dB
Isolation (1 Ghz)	−50 dB	−33 dB	−29.5 dB
Ports type	Absorptive	Reflective	Reflective
Ports number	4	4	3
Compression P1dB	30 dBm	5 dBm	10.6 dBm
Semiconductor technology	0.13 µm UltraCMOS	0.35 µm SiGe BiCMOS	0.35 µm CMOS
Package	QFN32 5 × 5 mm	QFN32 5 × 5 mm	-

* Parameters measured at 1.5 GHz.

## Data Availability

Not applicable.
